# Herpes simplex virus type 2 tegument protein UL56 relocalizes ubiquitin ligase Nedd4 and has a role in transport and/or release of virions

**DOI:** 10.1186/1743-422X-6-168

**Published:** 2009-10-16

**Authors:** Yoko Ushijima, Fumi Goshima, Hiroshi Kimura, Yukihiro Nishiyama

**Affiliations:** 1 Department of Virology, Nagoya University Graduate School of Medicine, 65 Tsurumai-cho, Showa-ku, Nagoya 466-8550, Japan

## Abstract

**Background:**

The ubiquitin system functions in a variety of cellular processes including protein turnover, protein sorting and trafficking. Many viruses exploit the cellular ubiquitin system to facilitate viral replication. In fact, herpes simplex virus (HSV) encodes a ubiquitin ligase (E3) and a de-ubiquitinating enzyme to modify the host's ubiquitin system. We have previously reported HSV type 2 (HSV-2) tegument protein UL56 as a putative adaptor protein of neuronal precursor cell-expressed developmentally down-regulated 4 (Nedd4) E3 ligase, which has been shown to be involved in protein sorting and trafficking.

**Results:**

In this study, we visualized and characterized the dynamic intracellular localization of UL56 and Nedd4 using live-cell imaging and immunofluorescence analysis. UL56 was distributed to cytoplasmic vesicles, primarily to the trans-Golgi network (TGN), and trafficked actively throughout the cytoplasm. Moreover, UL56 relocalized Nedd4 to the vesicles in cells transiently expressing UL56 and in cells infected with HSV-2. We also investigated whether UL56 influenced the efficiency of viral replication, and found that extracellular infectious viruses were reduced in the absence of UL56.

**Conclusion:**

These data suggest that UL56 regulates Nedd4 and functions to facilitate the cytoplasmic transport of virions from TGN to the plasma membrane and/or release of virions from the cell surface.

## Background

The ubiquitin system is a key regulatory mechanism for a variety of cellular processes: protein turnover, protein sorting and trafficking, signal transduction and cell-cycle control [1]. Ubiquitination is executed by a hierarchical cascade of three types of enzymes: ubiquitin-activating enzymes (E1s), ubiquitin-conjugating enzymes (E2s), and ubiquitin ligases (E3s) [2]. The human genome encodes more than 600 putative E3 ligases [3], which primarily provide substrate specificity. There are two main groups of E3 ligases: really interesting novel genes (RING) and homologous to E6AP carboxyl terminus (HECT) proteins. The neuronal precursor cell-expressed developmentally down-regulated 4 (Nedd4) family, comprised of nine members, is one of the main HECT E3 protein families. They are characterized by a unique domain architecture, with an amino-terminal C2 domain, two to four protein-protein interacting WW domains and a carboxyl terminal catalytic HECT domain [4].

Viruses depend heavily on functions provided by their host cells as intracellular parasites, and as such, have evolved diverse strategies to exploit the biology and biochemistry of hosts for their benefits. The ubiquitin system is one of the mechanisms exploited by many viruses; it is involved in viral assembly and release, viral transcriptional regulation, viral immune invasion and the suppression of apoptosis [5,6]. Regarding viral assembly and release, several Nedd4 family E3 ligases act to link the endosomal sorting complex required for transport (ESCRT) system and viral proteins [7]. The ESCRT system helps to sort cargo into intraluminal vesicles (ILVs) of multivesicular bodies (MVBs), a type of endosomes, and might also participate in the biogenesis of MVBs [8]. In fact, the ESCRT system is reportedly exploited by many enveloped RNA and DNA viruses [9].

Some viruses encode their own E3 ligases, de-ubiquitinating enzymes (DUBs) and adaptor proteins to modify the host's ubiquitin system [5,6]. Herpes simplex virus (HSV) encodes a ubiquitin ligase (ICP0) [10,11] and a DUB (UL36) [12]. In addition to these two proteins, the HSV type 2 (HSV-2) tegument protein UL56 was identified as a putative adaptor protein of Nedd4 E3 ligase [13]. Nedd4 is phosphorylated and degraded in wild-type HSV-2-infected cells in a UL56-dependent manner. UL56 interacts with Nedd4 and increases the ubiquitination of Nedd4, however UL56 itself is not ubiquitinated. Despite reports demonstrating interactions between UL56 and Need4, the role of this interaction in viral replication remains unclear.

HSV is a large, enveloped, double-stranded-DNA virus, which can cause various mild and life-threatening diseases, including herpes labialis, genital herpes, keratitis, encephalitis and neonatal herpes [14]. The HSV genome encodes at least 74 genes [15,16]. Approximately half of the genes are accessory genes: genes not essential for viral replication in cell-culture system [14]. The HSV accessory gene *UL56*, or a homologue, is encoded by most members of the *Alphaherpesvirinae *family [15-29]. Interestingly, HSV type 1 (HSV-1) *UL56 *has been shown to play an important role in pathogenicity *in vivo *[30,31], although little is known about its molecular mechanisms. HSV-2 UL56 is a 235-amino acid, carboxyl-terminal anchored, type II membrane protein that is predicted to be inserted into the viral envelope so that the amino-terminal domain is located in the virion tegument [32]. In this topology, UL56 is predicted to have a 216-amino acid cytoplasmic domain containing three PY motifs, which are important for its interaction with Nedd4 E3 ligase. UL56 has also been shown to associate with two other proteins: KIF1A [33], the neuron-specific kinesin; and HSV-2 UL11 [34], a tegument protein that has dynamic membrane-trafficking properties [35]. It is also involved in the envelopment and egress of viral nucleocapsids [36]. These interactions suggest that UL56 may be involved in vesicular transport in neurons, or viral envelopment and egress, however, the role and function of UL56 in viral replication and pathogenicity are still unknown.

In this study, to elucidate the biological role and function of HSV-2 UL56, and its interaction with E3 ligase Nedd4, we visualized and characterized the dynamic intracellular localization of UL56 and Nedd4 using live-cell imaging and immunofluorescence analysis. Furthermore, we investigated whether UL56 influenced the efficiency of viral replication by comparing growth properties of wild-type HSV-2 with those of UL56-deficient HSV-2.

## Results

### UL56 shows dynamic localization and relocalizes Nedd4

**Figure 1:**
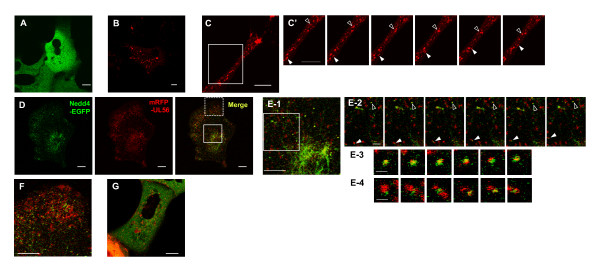
**UL56 shows dynamic localization and relocalizes Nedd4**. Images of live cells transiently expressing Nedd4-GFP and/or mRFP-UL56. Time-lapse images were captured with confocal microscopy. (A) Nedd4-GFP is diffusely distributed in the cytoplasm. (B-C') mRFP-UL56 is distributed in a vesicular pattern in the cytoplasm. (C) Images of mRFP-UL56 in the protruding portion of the cell. (C') Six sequential images of the area boxed in C. mRFP-UL56 moves bi-directly; filled arrowheads indicate puncta moving in plus-end direction, and open-arrowheads indicate puncta moving in minus-end direction. (D-F) Nedd4-GFP (shown in green) and mRFP-UL56 (shown in red) colocalizes and partially co-migrated. (E) Magnifications of the perinuclear region. (E-1) Magnification of the area boxed with solid lines in D. (E-2) Six sequential images of the area boxed in E-1. Arrow heads indicate punctuate structures containing both Nedd4-GFP and mRFP-UL56 with low motility (open arrow heads) or high motility (filled arrow heads). (E-3, -4) The punctum indicated with an open arrowhead (E-3) or that indicated with a filled arrowhead (E-4) is magnified. Overlaps between Nedd4-GFP- and mRFP-UL56-localization were not stable. (F) Magnification of the peripheral area boxed with dashed lines in D. Puncta positive for mRFP-UL56 alone and those positive for both mRFP-UL56 and Nedd4-GFP were more abundant in the peripheral of the cytoplasm. Scale bars: 10 μm, in A -E2, F and G; 2 μm, in E3 and E4.

We first explored the dynamics of Nedd4 and UL56 localization using live cell confocal microscopy. Nedd4 carboxyl-terminally tagged with EGFP (Nedd4-EGFP) and/or UL56 amino-terminally tagged with mRFP (mRFP-UL56) were transiently expressed in cells to visualize their distribution and movement. As previously observed in fixed cells, Nedd4-EGFP was diffusely distributed in the cytoplasm (Fig. 1A; additional file 1 [movie 1]) cells [13]. mRFP-UL56 was detected in a vesicular pattern and the puncta moved around the cytoplasm (Fig. 1B; additional file 2 [movie 2]). mRFP-UL56 puncta varied in size and moved in the different directions and at the different speeds. Fig. 1C shows frames of a time-lapse movie of mRFP-UL56 trafficking in the protruding portion of the cell (Fig. 1C; additional file 3 [movie 3]). Puncta displayed rapid movements in both the minus- and plus-end-directions, sometimes interrupted by stationary periods, and could be observed to merge, separate or change direction. The accumulation of puncta in the tip of the protrusion indicated a mild preference for plus-end-directional movement. mRFP-UL56AY, mutant UL56 with mutations of all three PY-motifs (PPXY to AAXY), showed similar distribution and movement to those of mRFP-UL56 (data not shown).

**Figure 2:**
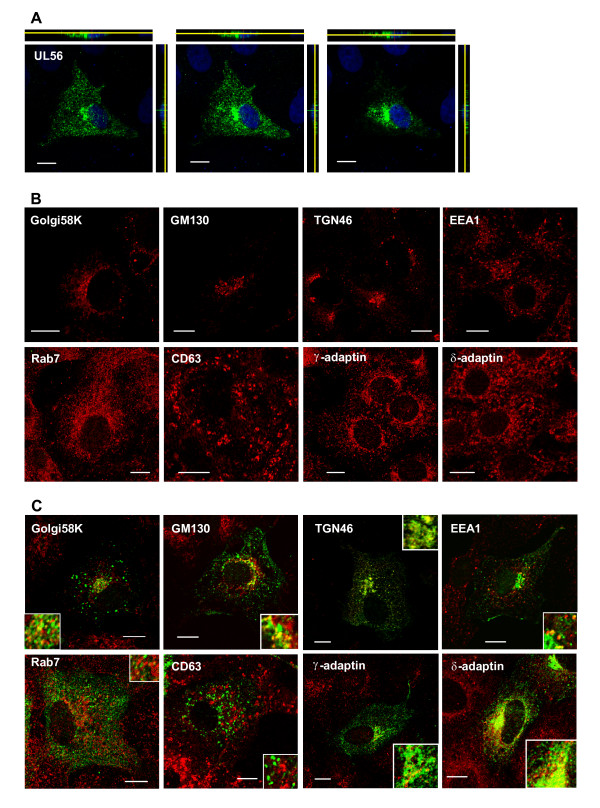
**UL56 localizes to cytoplasmic vesicular structures in transiently UL56-expressing cells**. Transiently UL56-expressing (A, C) or UL56-non-expressing cells (B) were immunostained for UL56 (shown in green) and/or marker proteins for Golgi, trans-Golgi network (TGN) or endosomes (shown in red). (A) UL56 is distributed the cytoplasm in a vesicular pattern with accumulation in the perinuclear region. The serial confocal sections of UL56-expressing cells in the x-y plane with x-z (top panels) and y-z (right panels) projections are shown. X-z sections are shown with the apical side down; and y-z sections are shown with the apical side to the left. Yellow lines indicate the z-levels of x-y sections. Nuclear DNA was stained with DRAQ5 (shown in blue). (B) Every protein marker showed its specific distribution. (C) UL56 colocalized predominantly with marker proteins for TGN (TGN46, γ-adaptin and δ-adaptin), and partially with marker proteins for Golgi complex proteins (Golgi58K and GM130) and early endosomes (EEA1). Scale bars, 10 μm.

**Figure 3:**
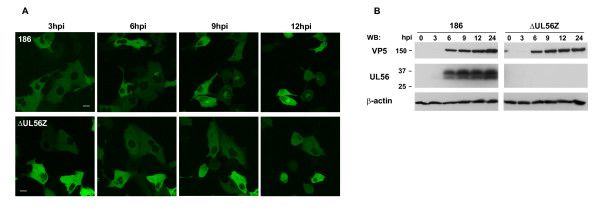
**Nedd4 accumulates in the perinuclear region in cells infected with wild-type HSV-2 but not in cells infected with UL56-deficient HSV-2**. (A) Images of live cells transfected with a Nedd4-GFP expressing plasmid and sequentially infected with wild-type (186) (top panels) or UL56-deficient (ΔUL56Z) viruses (bottom panels). Nedd4-EGFP accumulated in the perinuclear region after 6 h postinfection (hpi) in cells infected with wild-type viruses, but remained diffuse in cells infected with ΔUL56Z. Scale bars, 10 μm. (B) Western blots of cell lysates infected with wild-type (186) or ΔUL56Z viruses. UL56 was detected after 6 h postinfection in cells infected with wild-type viruses. VP5, a major capsid protein, was detected after 6 h postinfection at equivalent levels in cells infected with wild-type viruses or ΔUL56Z. β-actin was used as a loading control.

**Figure 4:**
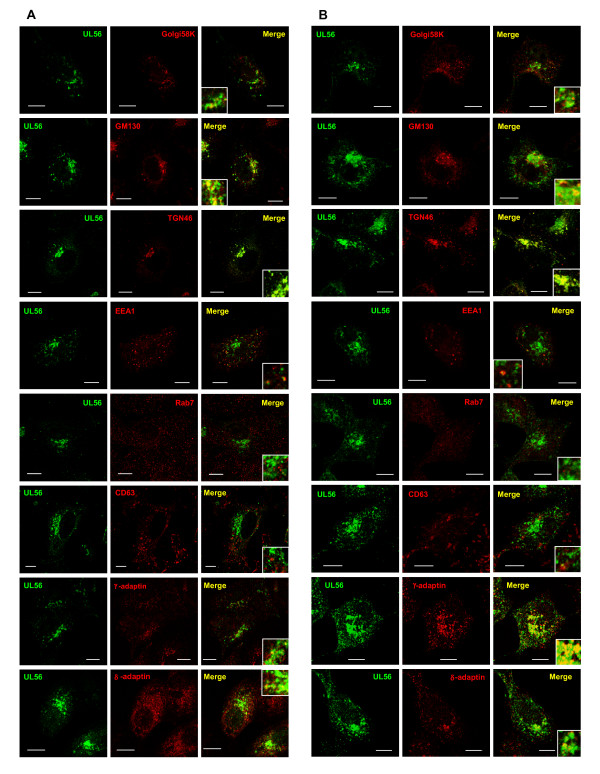
**UL56 localizes to cytoplasmic vesicular structures in HSV-2 infected cells**. Cells infected with wild-type viruses were fixed at 6 h postinfection (A) or 9 h postinfection (B), and immunostained as described in Fig. 3. UL56 (shown in green) accumulated in the perinuclear region with vesicular distribution in the cytoplasm. UL56 predominantly colocalized with marker proteins for TGN (TGN46, γ-adaptin and δ-adaptin), and partially with marker proteins for Golgi complex proteins (Golgi58K and GM130) and early endosomes (EEA1) (shown in red). Little or no overlap was detected between UL56 and either rab7 or CD63. Scale bars, 10 μm.

Next, we investigated whether UL56 alters the localization of Nedd4. Coexpression of mRFP-UL56 with Nedd4-EGFP markedly reduced the EGFP-signal but not the mRFP-signal (Fig. 1D), suggesting that the stability of Nedd4-EGFP was changed in the presence of mRFP-UL56. mRFP-UL56 was distributed in a vesicular pattern, similar to cells expressing mRFP-UL56 alone. Moreover, the subcellular distribution of Nedd4-GFP was markedly changed in the presence of mRFP-UL56, such that Nedd4-GFP now showed a vesicular distribution and colocalized with mRFP-UL56, and in some instances showed a filamentous distribution near the nucleus (Fig. 1E-1). A substantial portion of Nedd4-GFP in the vesicular pattern colocalized with mRFP-UL56, while only small portion of mRFP-UL56 colocalized with Nedd4-GFP. As was the case for mRFP-UL56 singly expressing cells, the majority of puncta positive only for mRFP-UL56 displayed continuous movement in cells that coexpressed Nedd4-GFP. Most punctuate structures positive for both Nedd4-GFP and mRFP-UL56 were less motile than those with mRFP alone (Fig. 1E-2, open arrowheads). Some punctuate structures with both proteins moved as fast as mRFP-UL56 puncta (Fig. 1E-2, filled arrowheads). Overlap between the localization of Nedd4-GFP and mRFP-UL56 was not stable; the pattern of overlap changed rapidly, and even merge or separation of the two proteins was observed (Fig. 1E-3 and 1E-4). Nedd4-GFP and a part of mRFP-UL56 colocalized to punctate structures also in the peripheral region of the cytoplasm (Fig. 1F); the punctuate structures showed similar kinetics to those of puncta near the nucleus.

On the contrary, Nedd4-GFP, when co-expressed with mRFP-UL56AY, remained largely diffuse in the cytoplasm. Only several Nedd4-GFP puncta were detected and they colocalized with mRFP-UL56AY (Fig. 1G). We have previously reported that Nedd4 colocalizes with UL56 but only partially with UL56AY in fixed cells [13], which is consistent with these observations. Live cell imaging showed more clearly the difference of Nedd4 distribution between in UL56- and in UL56AY-expressing cells than immunofluorescence analysis of fixed cells.

### UL56 localizes to the Golgi complex, trans-Golgi network and early endosomes in cells transiently expressing UL56

We next sought to determine detailed intracellular localization of UL56 using immunostaining. UL56 was distributed throughout the cytoplasm in a vesicular pattern with accumulation in the perinuclear region (Fig. 2A), consistent with previous observations [13,32]. In order to identify the punctuate structures localized by UL56, cells transiently expressing UL56 were stained for Golgi-, trans-Golgi network (TGN), or endosomal marker proteins. Transient expression of UL56 caused no apparent change in the localization of marker proteins except rab7; rab7 was concentrated in the perinuclear space to a greater extent in UL56-expressing cells (Fig. 2B and 2C). UL56 only partially colocalized with Golgi58K, a marker for the Golgi complex [37], and GM130, a marker for *cis*-Golgi [38]. In contrast, a substantial portion of UL56 colocalized with TGN46, a marker for TGN [39,40], suggesting that UL56 predominantly localizes to TGN. We also tested for constitutitve proteins of the coated vesicle adaptor protein complex (AP): γ-adaptin, a marker for AP-1 [41]; δ-adaptin, a marker for AP-3 [42]. Both AP-1 and AP-3 localizes to TGN and endosomes, with AP-3 localizes more to endosomes [43]. UL56 colocalized with both γ-adaptin and δ-adaptin, albeit greater colocalization with δ-adaptin, suggesting that UL56 localizes to the endosomal compartment as well as TGN. As expected, UL56 partially colocalized with EEA1, a marker for early endosomes [44]. UL56 did not colocalize with rab7, a marker for late endosomes [45], or CD63 [46,47], a marker for late endosomes/MVBs. These data suggest that UL56, if expressed alone, is predominantly present in TGN and partially in Golgi complex and early endosomes.

### HSV-2 infection causes accumulation of Nedd4 in the perinuclear region in a UL56-dependent manner

We next explored the dynamics of Nedd4 in HSV-2 infected cells by live cell confocal microscopy. Cells transiently expressing Nedd4-EGFP were infected with wild-type HSV-2 (strain186) or UL56-deficient HSV-2 (ΔUL56Z). In cells infected with wild-type HSV-2, Nedd4-EGFP remained diffuse in the cytoplasm in the early-phase of viral replication (3 h postinfection), as shown in Fig. 3A (top panels). The localization began to change around 5-6 h postinfection, such that Nedd4-EGFP accumulated in the perinuclear region. On the contrary, Nedd4-GFP remained diffuse throughout the analysis period (2-24 h postinfection) in ΔUL56Z-infected cells (Fig. 3A, bottom panels). These observations suggest that HSV-2 infection causes Nedd4 to accumulate in the perinuclear region in a UL56-dependent manner. In addition, UL56 was detected after 6 h postinfection in wild-type HSV-2 infected cells with Western blot analysis (Fig. 3B) or immunofluorescence analysis (Fig. 4A). Temporal coincidence between the change of Nedd4 distribution and UL56 expression in HSV-2 infected cells further supports this view Nedd4 and UL56 colocalization.

### UL56 localizes predominantly to trans-Golgi network in HSV-2 infected cells

We further investigated detailed intracellular localization of UL56 in infected cells using immunostaining. Infected cells contain abundant viral proteins which are incorporated into cells upon infection or newly synthesized during infection. Viral proteins interact with both viral and cellular proteins, thus other viral proteins can influence UL56 distribution directly or indirectly.

UL56 accumulated in the perinuclear region with vesicular distribution in the cytoplasm at 6 h postinfection (Fig. 4A), and increased both in the perinuclear region and in the peripheral region at 9 h postinfection (Fig. 4B). The pattern of the intracellular distribution of UL56 in infected cells was similar to that in cells transiently expressing UL56. However, UL56 accumulated more distinctly in the perinuclear region and spread to lesser extent in the peripheral region in infected cells. UL56 colocalized partially with Golgi58K and GM130 in the perinuclear region, and predominantly with TGN46. Infections changed distributions of some marker proteins. Golgi58K and GM130 were in part dispersed around the perinuclear region in infected cells. This finding is consistent with the previous report that the Golgi apparatus becomes disorganized and distorted in infected cells [32]. TGN was detected in the cytoplasm with a vesicular pattern besides the perinuclear region. Partial colocalization of UL56 was also seen with γ-adaptin, δ-adaptin, and EEA1, whereas UL56 showed little or no colocalization with markers for late endosomes. UL56 and rab7 did not colocalize either at 6 h or 9 h postinfection. The overlap of UL56 with CD63 was not detected at 6 h postinfection, but detected in only a few vesicles around the perinuclear region at 9 h postinfection. HSV-2 glycoprotein G (gG), an envelope protein, did not colocalized with CD63 at 6 h or 9 h postinfection (data not shown). These observations demonstrate that in infected cells, UL56 localized predominantly to TGN, and partially to Golgi complex and early endosomes, but not to late endosomes/MVBs.

### Nedd4 and UL56 colocalizes predominantly to trans-Golgi network in HSV-2 infected cells

**Figure 5:**
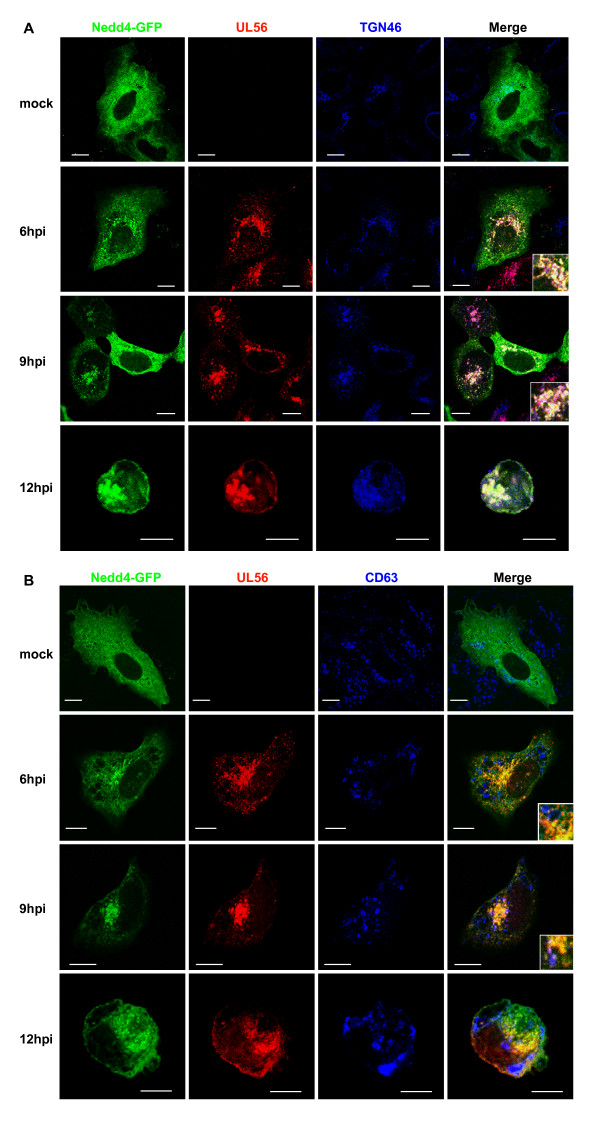
**Nedd4 and UL56 colocalized to trans-Golgi network in HSV-2 infected cells**. Cells were transfected with a Nedd4-GFP expressing plasmid, subsequently infected with wild-type HSV-2, fixed at indicated times postinfection, immunostained for UL56 and either TGN46 (A) or CD63 (B). (A) Nedd4-GFP (shown in green) and UL56 (shown in red) substantially colocalizes to TGN46 (shown in blue). (B) Nedd4-GFP and UL56 shows no (at 6 or 12 h postinfection [hpi]) or little (at 9 h postinfection) colocalization with CD63 (shown in blue). Scale bars, 10 μm.

We then determined the localization of Nedd4 in infected cells. Nedd4-GFP showed diffuse distribution throughout the cytoplasm. Infection caused Nedd4-GFP to accumulate in the perinuclear region, and in addition, Nedd4 was also distributed in a vesicular pattern in the peripheral of the cytoplasm. The distribution pattern of UL56 in cells transiently expressing Nedd4-GFP was not different from that in Nedd4-GFP-non-expressing cells. Nedd4-GFP markedly colocalized with UL56 and TGN46 after 6 h postinfection (Fig. 5A). However, Nedd4-GFP showed little colocalization with CD63; Nedd4-GFP, similar to UL56, colocalized with CD63 in very few vesicles only at 9 h postinfection (Fig. 5B). If HSV-2 exploited MVBs and/or late endosomes for assembly or release of virions, and if Nedd4 and UL56 were involved in the process, a greater amount of Nedd4 and UL56 should colocalize with CD63 as the infection proceeds. However, little colocalization was observed between CD63 and either UL56 or Nedd4 even at 12 h postinfection.

### Extracellular infectious viruses are decreased in the absence of UL56

**Figure 6:**
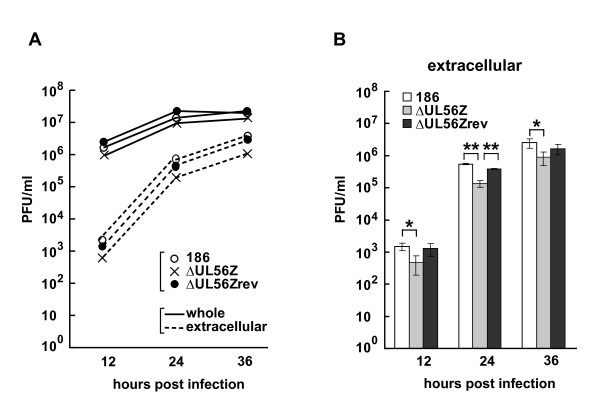
**Extracellular infectious viruses are decreased in the absence of UL56**. Single-step growth analysis of 186 (wild-type), ΔUL56Z and ΔUL56Zrev viruses. Vero cells were infected with viruses at an MOI of 3 PFU/cell, and harvested at indicated times postinfection; culture medium and cells were analyzed together to determine 'whole' viral yields, or only culture medium were analyzed to determine 'extracellular' viral yields. (A) The results from one representative experiment are shown. (B) Results of extracellular viral yields from three independent experiments are shown as mean ± standard deviation. Extracellular ΔUL56Z showed statistically significant decreases compared to wild-type viruses. *p < 0.05, **p < 0.01; two-tailed t-test.

We have reported that UL56-deficient virus (ΔUL56Z) shows no apparent growth defect compared to wild-type HSV-2 (186) in Vero cells and SK-N-SH cells (human neuroblastoma cells) [13]. In the previous study, we analyzed single-step growth kinetics of 'whole' viruses, which contained both extracellular- and intracellular-infectious virions. However, it is possible that UL56 plays a role in the intracellular transport and/or release of virions after formation of infectious virions. We thus investigated single-step growth kinetics of extracellular viruses in this study, and found that extracellular ΔUL56Z showed statistically significant decreases in titers compared to wild-type viruses: at 12 h postinfection there was a 67.9% decrease, p = 0.02; at 24 h postinfection there was a 75.2% decrease, p < 0.001; at 36 h postinfection there was a 64.1% decrease, p = 0.04 (Fig. 6). In contrast, regarding the 'whole' viruses, there was no statistically significant difference in growth between wild-type virus and ΔUL56Z. These data suggest that, although infectious virions were produced in cells infected with ΔUL56Z as efficiently as in those with wild type virus, virions were not efficiently transported and/or released from the cells infected with ΔUL56Z.

## Discussion

The present study demonstrates that HSV-2 tegument protein UL56 was distributed to cytoplasmic vesicles, primarily to TGN and partially to Golgi and early endosomes, and trafficked actively throughout the cytoplasm. Moreover, UL56 relocalized the E3 ligase Nedd4 to the vesicles both in cells transiently expressing UL56 and in cells infected with HSV-2. We have also demonstrated that the amount of extracellular infectious viruses were reduced in the absence of UL56, suggesting a role for UL56 in the transport and/or release of virions.

The observation of vesicular distribution and active trafficking of UL56 supports the hypothesis that UL56 is involved in vesicular transport [13,32-34]. UL56 relocalized Nedd4, a cytosolic E3 ligase, to vesicles. The unstable overlap between the localization of UL56 and that of Nedd4 suggests that the interaction of the two proteins is dynamic and transient. Given that Nedd4 participates in many cellular trafficking activities including protein sorting and viral budding, UL56 can regulate the function of Nedd4 by recruiting Nedd4 to substrates, without affecting its activity. Interestingly, Nedd4 family-interacting protein 2 (NDFIP2, N4WBP5A), a regulatory protein of Nedd4 family members, has been reported to relocalize Nedd4 to vesicular structures [48]. NDFIP1 [49] and NDFIP2 (NDFIPs) [50,51], and UL56 have several characteristics in common: transmembrane domains; three cytoplasmic PY motifs; interact with Nedd4; enhance the ubiquitination of Nedd4. Although NDFIPs are involved in membrane trafficking through MVB machinery [52,53], UL56 showed no apparent localization to late endosomes/MVBs.

Newly synthesized HSV nucleocapsids exit from the nucleus by budding at the inner nuclear membrane and subsequently translocate into the cytoplasm by fusion of the primary envelope with the outer nuclear membrane. In the cytoplasm, nucleocapsids acquire additional teguments and then obtain their final envelope by budding into cytoplasmic vesicles [54]. TGN has been shown to be involved the generation of the final virion envelope [55-57]. Primary localization of UL56 to TGN suggests that UL56, a tegument protein which is predicted to be inserted the envelope, is incorporated into virions at TGN. Trafficking of virions from TGN to the cell surface has not yet been elucidated. The MVB pathway is proposed as a possible pathway, because ESCRT machinery has been shown to facilitate HSV-1 final envelopment [58]. In the present study, either UL56 or gG showed little or no localization to late endosome/MVBs. Therefore, our finding does not support the view that HSV uses the MVB pathway, but is consistent with a report that no HSV-1 particles are observed in MVBs [59]. HSV might exploit the ESCRT system in a different way from that of cells. In fact, UL56 could be associated with ESCRT machinery in TGN, given that Nedd4 links viral proteins and the ESCRT system. Further investigation is needed to clarify the association between the ESCRT system and HSV-2 infection.

**Figure 7:**
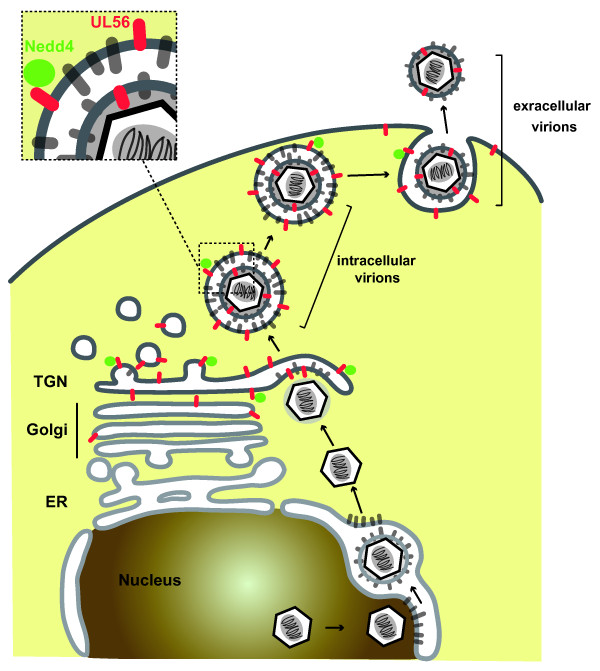
**A model of maturation, transport and release of HSV-2 virions**. After exit from the nucleus, nucleocapsids in the cytoplasm acquire additional teguments and obtain the final envelope by budding into trans-Golgi network (TGN). Vesicles containing virions are transported to the cell surface and release virions by fusion with the plasma membrane. In the TGN, UL56 is incorporated into virions or remains in the limiting membrane of the vesicles. UL56 protrudes into the cytoplasm from the membrane of vesicles containing virions, and interacts with proteins which are involved in membrane trafficking, transport or membrane fusion. This interaction facilitates the transport and/or release of virions. Nedd4 can play some role in this process via its interaction with UL56.

The growth kinetics of UL56-deficient virus gave rise to the view that UL56 functions in the transport and/or release of virions. UL56 is predicted to leave 216 amino acids out of 235 total amino acids in the tegument layer of virions and in the cytoplasm in infected cells (Fig. 7). Active trafficking of transiently expressed UL56, which represents UL56 inserted into vesicular membranes in infected cells, leads us to propose the following model for UL56 function in viral replication. UL56 protruding from the limiting membrane of virion-containing vesicles to the cytoplasm interacts with other cellular and/or viral proteins, which are involved in membrane trafficking, transport or membrane fusion. This interaction facilitates the transport and/or release of virions. Nedd4 may also be involved in this process. In addition, our data on the growth kinetics of UL56-deficient virus do not exclude the possibility that UL56 functions in envelopment of nucleocapsids, because redundant viral proteins can cover some defects of UL56-deficient virus. The mechanism of tegumentation, final envelopment of nucleocapsids, transport and release of virions remains unclear despite many attempts, partially due to the high redundancy of viral proteins [60].

## Conclusion

This study provides new insights into the function of HSV-2 UL56 in regulating E3 ligase Nedd4 and also in viral replication. How the regulation of Nedd4 by UL56 functions in HSV-2 infection remains unclear but warrants further investigation. Studies on the interaction between Nedd4 and UL56 will help to clarify both cellular process and viral pathogenesis.

## Methods

### Cells and viruses

Vero cells (African green monkey kidney cells) were obtained from the RIKEN BioResource Center (Ibaraki, Japan) and used through out this study. Vero cells were maintained in Eagle's minimum essential medium (MEM) supplemented with 8% calf serum (CS), 100 U/ml penicillin and 100 μg/ml streptomycin. The HSV-2 wild-type strain 186, the UL56-deficient recombinant virus based on strain 186 (ΔUL56Z) [34] and the UL56-reverted virus based on ΔUL56Z (ΔUL56Zrev) [13] were used in this study. Viruses were propagated in Vero cells by infection at low multiplicity of infection (MOI) (0.01 PFU/cell), and infected cells and growth medium were harvested together when almost all cells showed cytopathic effects. After a cycle of freezing and thawing, supernatants were cleared of cell debris by centrifugation at 3000 rpm for 5 min at 4°C and stored at -80°C as virus stocks. Titers of virus stocks were determined on Vero cells by plaque assay.

### Expression vectors

The Nedd4 ORF was PCR amplified from pFLAG-Nedd4 [13] and cloned into pEGFP-N3 (Clontech, Mountain View, CA) to generate pNedd4-EGFP. The ORF of UL56 and UL56AY, mutant UL56 with all three PPXY motifs mutated to AAXY (P23A, P24A, P49A, P50A, P145A, P146A), were PCR amplified from pcDNA-UL56 and pcDNA-UL56AY, respectively, and cloned into pcmRFP [61] to generate pcmRFP-UL56 and pcmRFP-UL56AY. The expression of fusion proteins in cells transfected with these plasmids were verified using western blotting: with anti-Nedd4 and anti-GFP antibodies, for Nedd4-GFP; and anti-UL56 and anti-RFP antibodies, for UL56 (data not shown).

### Transfection and infection

Cells were plated in 35-mm dishes and incubated for 24 h before transfection or infection. In transfection experiments, 1 μg of each plasmid was transiently transfected into cells using Lipofectamine 2000 (Invitrogen, Carlsbad, CA) following the manufacturer's recommendations. In some experiments, transfected cells were further infected with HSV-2 48 h posttransfection. Infections were performed by exposing cells to a minimal volume of virus diluted at an MOI of 3 PFU/cell in MEM without CS. After a 1 h adsorption period, the virus inocula was replaced with MEM containing 5% CS, and cells were incubated for indicated time period.

### Live cell confocal microscopy

Time lapse confocal imaging of live cells was performed as previously described [61] using Zeiss LSM510 system (Carl Zeiss, Oberkochen, Germany). In transfection experiments, cells were transfected with the indicated expression plasmids and incubated for 48 h. The recording was made at 0.63 Hz (one frame every 1.58 sec; for supplemental movies 1 and 2) or at 1.01 Hz (one frame every 0.99 sec; for supplemental movie 3) for 120 images. Images were sequenced to generate the movie, and converted into Microsoft Audio/Video Interlaced format with the LSM software. Each movie was then compressed and converted intoQuickTime format using QuickTime software (Apple, Cupertino, CA). In infection experiments, cells transfected with pNedd4-EGFP were further infected with HSV-2 186 or ΔUL56Z. Imageswere captured from 2 to24 h postinfection every 12 min. 24 *z*-axis confocal sections were obtained at 0.5-0.6 μm steps at every time point; and the images were projected onto a single plane. Projected time-lapse images were processed in the same way as in transfection experiments.

### Immunofluorescence confocal microscopy

Indirect immunofluorescence confocal microscopy was performed as previously described [13] with slight modifications. In brief, cells grown on cover slips were fixed in 4% paraformaldehyde in PBS for 15 min and permeabilized with 0.1% Triton X-100 for 5 min at room temperature. Coverslips were incubated for 1 h at room temperature sequentially with 20% normal goat serum (DAKO, Glostrup, Denmark), primary and secondaryantibodies. The following were used as primary antibodies: polyclonal anti-UL56 (1:200 dilution) [32] and -TGN46 (1:100; AbD Serotec, Oxford, UK) antibodies, monoclonal anti-Golgi58K (clone 58K-9; 1:50; SIGMA, Saint Louis, MO), -GM130 (clone 35; 1:20; BD Transduction Laboratories, Franklin Lakes, NJ), -adaptin γ (clone 88; 1:100; BD), -adaptin δ (clone 18; 1:50; BD), -EEA1 (clone 14; 1:100; BD), -rab7 (clone Rab7-117; 1:50; SIGMA), and -CD63 (clone H5C6; 1:200; BD) antibodies. AlexaFluor 488- or AlexaFluor 647- conjugated goat anti-rabbit, AlexaFluor 546- or AlexaFluor 647- conjugated goat anti-mouse, and AlexaFluor 546-conjugated goat anti-sheep IgG (1:500; Invitrogen) were used as secondary antibodies. In some experiments, coverslips were additionally incubated with 1.25 μM DRAQ5 (Biostatus, Leicestershire, UK) for 15 min at room temperature to stain nuclear DNA. Confocal images were captured using Zeiss LSM510 system (Carl Zeiss).

### Extraction of cell lysates and Western blot analysis

All procedures were performed as previously described [13]. The following were used as primary antibodies: a polyclonal anti-UL56 antibody (1:5000 dilution) [32], monoclonal anti-VP5 (clone3B6; 1:3000; Abcam, Cambridge, UK) and -β-actin (clone AC-15; 1:5000; SIGMA) antibodies.

### Viral replication kinetics assay

Cells in 35-mm dishes were infected with wild-typeHSV-2 (186), ΔUL56Z or ΔUL56Zrev mutants at an MOI of 3. After 1 h of adsorptionat 37°C, the inoculum was replaced with 1 ml citrate buffer (40 mM citric acid, 135 mM NaCl, 10 mM KCl [pH3.0]) and incubated at room temperature for 2 min to inactivate viruses which had not penetrated the cell. The cells were then washed twice with PBS, and MEM containing 5% CS was addedto each dish. At the indicated time pointspostinfection, for 'whole' samples, culture medium and cells were frozen together and thawed once, and then supernatants were cleared of cell debris by centrifugation at 3000 rpm for 5 min. For 'extracellular' samples, culture medium were cleared by centrifugation at 3000 rpm for 5 min at 4°C, and frozen and thawed once. The viruses in the supernatants were titrated on Vero cells. Statistical analysis was performed using two-tailed t-test.

## Competing interests

The authors declare that they have no competing interests.

## Authors' contributions

YU performed the experimental work, conducted the data analysis and drafted the manuscript. FG and HK participated in the data analysis and review of the manuscript. YN performed project planning, participated in the data analysis and helped to draft the manuscript. All authors read and approved the final manuscript.

## Supplementary Material

Additional file 1 **Live imaging of cells expressing Nedd4-EGFP**. Supplement to Fig. 1A. Nedd4-GFP is diffusely distributed in the cytoplasm. Movie is ×20 accelerated relative to real time. Click here for file

Additional file 2 **Live imaging of cells expressing mRFP-UL56**. Supplement to Fig. 1B. Actively moving structures were seen in the cytoplasm. Movie is ×20 accelerated relative to real time. Click here for file

Additional file 3 **Live imaging of mRFP-UL56 in the protruding portion of the cell**. Supplement to Fig. 1C. Actively moving structures were seen. They showed rapid movements in both the minus- and plus-end-directions, sometimes fused and split. Movie is ×20 accelerated relative to real time. Click here for file
